# Market participation, household food security, and income: The case of cowpea producers in northern Nigeria

**DOI:** 10.1002/fes3.211

**Published:** 2020-06-02

**Authors:** Julius Manda, Arega D. Alene, Adane Hirpa Tufa, Shiferaw Feleke, Tahirou Abdoulaye, Lucky O. Omoigui, Victor Manyong

**Affiliations:** ^1^ International Institute of Tropical Agriculture Arusha Tanzania; ^2^ International Institute of Tropical Agriculture Lilongwe Malawi; ^3^ International Institute of Tropical Agriculture Dar es Salaam Tanzania; ^4^ International Institute of Tropical Agriculture Bamako Mali; ^5^ International Institute of Tropical Agriculture Kano Nigeria

**Keywords:** cowpea, Cowpea market participation, food security, income, northern Nigeria, rural traders, urban traders

## Abstract

This article evaluates the impact of cowpea market participation on household food security and income in northern Nigeria. Using household survey data from a representative sample of over 1,500 farm households and applying a combination of instrumental variable techniques and dose–response functions, we found that cowpea market participation had a statistically significant positive impact on household food security and income. Cowpea market participation increased food expenditure by 1.6% and household income by 0.7% with a 10 unit increase in the quantity of cowpea sold. These results underscore the importance of cowpea market participation for household food security and income improvement. We also found that selling cowpea to rural and urban traders significantly increased household income, food expenditure, and food security. Results show that selling cowpea to rural and urban traders increased household income by 17% and 13%, respectively. The results point to the need for an enabling policy environment and public infrastructure to enhance market participation of farmers and traders. Public infrastructure investments in the form of feeder road construction and maintenance in the distant villages are encouraged, which in the long run can translate into improved cowpea productivity and welfare of smallholder farmers.

## Graphical abstract

This paper evaluates the impact of cowpea market participation on household food security and household income in northern Nigeria. We find that cowpea market participation has a positive and significant effect on household food security and income.

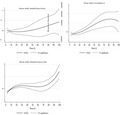

## 1 INTRODUCTION

Although there is some progress in the fight against hunger in the world through increase in food production, many people are still food insecure and suffer from some form of malnutrition (FAO, IFAD, & WFP, 2015; Godfray et al., 2010; Sibhatu, Krishna, & Qaim, 2015). Globally, an estimated 821 million people were undernourished in 2017, with the majority living in the developing countries (FAO et al., 2018). Sub‐Saharan Africa has the world's highest prevalence of undernourishment that was projected to be 23.2% during the same year (FAO et al., 2018). Nigeria remains a country with high levels of poverty, food insecurity, and malnutrition. An estimated 54%^1^ of Nigerians live on less than US$1.9 per day and about 37% of the children were malnourished in 2013 (NPC & ICF, 2014; World Bank, 2018). Northern Nigeria has the highest poverty and malnutrition rates (Amare, Benson, Fadare, & Oyeyemi, 2018; NBSN, 2010).

Smallholder market participation has long been touted as a strategy to improve farmers' productivity, income, food security, and poverty (Barrett, 2008; Radchenko & Corral, 2018; Sibhatu et al., 2015). However, no conclusive evidence exists on its effect on food security and nutrition. On the one hand, there are studies which have shown that commercialization of agricultural produce is effective in improving food security and nutrition (e.g., Muriithi & Matz, 2015; Ogutu, Gödecke, & Qaim, 2017; Ogutu, Gödecke, & Qaim, 2019; Ogutu & Qaim, 2019; Radchenko & Corral, 2018). On the other hand, studies such as Carletto, Corral, and Guelfi (2017) found little evidence that agricultural commercialization has a positive effect on the nutritional status of smallholder farmers. We contribute to this debate on market participation by examining the impact of cowpea market participation on household food security and income in northern Nigeria using rigorous econometric approaches, and a unique and comprehensive household‐level data collected from a nationally representative sample of over 1,500 cowpea producing households in the region. Understanding the relationship between cowpea (an important crop in the northern Nigeria) market participation and food security can have important policy implications.

Most previous studies on market participation have mainly concentrated on understanding the factors that facilitate or hinder incidence and level of market participation (e.g., Bellemare & Barrett, 2006; Burke, Myers, & Jayne, 2015; Mignouna, Abdoulaye, Akinola, Kamara, & Oluoch, 2016; Olwande, Smale, Mathenge, Place, & Mithöfer, 2015). Others have gone beyond determinants and examined the effects of market participation on income, food security, and poverty (Muriithi & Matz, 2015; Ogutu & Qaim, 2019; Radchenko & Corral, 2018). Yet, to our knowledge, none of these studies considered the associated benefits of different market choices (rural and urban) on food security and welfare of smallholder farmers, with the exception of a study by Montalbano, Pietrelli, and Salvatici (2018). In this study, a household was considered to have participated in the market if they sold any amount of cowpea.

We add to the growing literature on market participation in the following ways. First, unlike many previous studies on market participation which use the traditional discrete choice of market participation versus nonparticipation, this study uses a continuous variable, the quantity of cowpea sold, as an indicator of market participation. This enables us to measure the impact of the level or intensity of market participation on our indicators of food security (household dietary diversity [HDD] and food expenditure per capita) and household income per capita. Further, taking advantage of the continuous nature of our treatment variable, we assess the impact of cowpea market participation by estimating dose–response functions and their derivatives, the marginal treatment effects. Marginal treatment effects estimate the heterogeneous returns to market participation (Radchenko & Corral, 2018). Second, we examine the farmers' decisions of pursuing different market choices in the cowpea value chain. Specifically, we consider whether selling cowpea to rural traders or urban wholesalers/traders has a positive effect on HDD, food expenditure per capita (hereafter referred to as food expenditure), and household income per capita (hereafter referred to as household income). This is especially important in view of the scantiness of the literature on grain assembly in rural Africa, making it difficult for policymakers to better understand the effects of grain assembly on rural farm households (Sitko & Jayne, 2014). To achieve these objectives, we used instrumental variable (IV) techniques to a unique and comprehensive household‐level data involving a nationally representative sample of over 1,500 cowpea farmers in Nigeria. The IV technique allowed us to control for both observed and unobserved characteristics that would otherwise bias our results.

In summary, the main aim of the study was to examine the impact of cowpea market participation on household food security and income. Specifically, this study was done to understand the associated benefits of different market choices on food security and welfare of smallholder farmers. This information has particularly been lacking in the literature.

The rest of the article is organized as follows. The following section briefly describes the structure of the cowpea market in Nigeria, followed by the empirical framework and data collection procedure. In this section, we outline the empirical models used to estimate the impact of cowpea market participation on HDD, food expenditure, and household income. The penultimate section presents the empirical results and discussion, and Section 4 draws conclusions and describes the implications of the results for policy.

### 1.1 Cowpea marketing in Nigeria

Nigeria is the largest cowpea producer in the world as well as the largest consumer and importer of cowpea in Africa (Alene & Manyong, 2007; Langyintuo et al., 2003; Mishili et al., 2009). The crop is important for small‐scale farmers in terms of soil fertility management, and sources of cash income, high‐quality protein food, and fodder for animals (Kristjanson, Okike, Tarawali, Singh, & Manyong, 2005; Mishili et al., 2009; Singh, Ehlers, Sharma, & Filho, 2002).

The cowpea value chain^2^ comprises traders that ensure the movement of grain from rural markets to urban wholesale markets and finally to consumer markets (Mishili et al., 2009). Most of the cowpea production is sold as grain although some cowpeas are purchased as green pods at harvest time, and in some regions, the leaves are eaten as greens (Mishili et al., 2009). In most cases, farmers sell their marketable surplus cowpea either in small quantities to rural assemblers and commission agents, who aggregate them into 100 kg bags which are then sold to urban wholesalers or sometimes sold in large quantities directly to urban wholesalers (Langyintuo et al., 2003; Lowenberg‐DeBoer & Ibro, 2008; Mishili et al., 2009). The center for cowpea trade in Nigeria, which also happens to be the largest cowpea market in the world, is Dawanau market located in Kano state. The traders in Dawanau market support a well‐developed collection system throughout northern Nigeria where they provide credit to buyers in local markets. The local traders buy cowpea in small quantities, which are later aggregated and stored until transported to Kano (Lowenberg‐DeBoer & Ibro, 2008). A large quantity of cowpea sold by farmers in north central Nigeria pass through the Dawanau market where independent grain and cowpea traders operate (Langyintuo et al., 2003).

In this trading system, rural traders play a vital role in buying cowpea from markets in remote areas which may not be accessible by the bigger traders in the urban areas. Notwithstanding, most studies argue that rural traders usually exploit farmers by offering them low prices. For example, Sitko and Jayne (2014) noted that most people have the view that unreliable market access conditions compel farmers to sell their produce to village‐level grain assemblers who exploit their lack of formal markets by offering prices that are below the cost of production. In this study, we aim to assess whether selling to rural and urban traders influences the welfare of smallholder cowpea farmers in Nigeria.

## 2 MATERIALS AND METHODS

### 2.1 Empirical strategy

To assess the impact of cowpea market participation on HDD, food expenditure, and household income, we estimated the following linear equation

(1) $$Y_{i}=\beta_{0}+\beta_{1}T_{i}+\beta_{2}X_{i}+\varepsilon_{i}$$

where *Y_i_* represents the outcome variables (household dietary diversity scores [HDDS],^3^ food expenditure, and household income), *T_i_* is our treatment variable, market participation represented by the quantity of cowpea sold, *X_i_* is the vector of control variables, and *ε_i_* is the random disturbance term. The parameter of interest is *β_i_* which measures the effect of cowpea market participation on our outcome variables. Market participation can be modeled as

(2) $$T_{i}=\alpha_{0}+\alpha_{1}Z_{i}+\varepsilon_{i}$$

where *Z_i_* is a vector of the determinants of market participation . *T_i_* is, however, potentially endogenous such that the cov (*T_i_*, *ε_i_*) ≠ 0. To correct this, we employed the IV regression that accounts for unobserved characteristics, yielding unbiased and consistent estimates. This, however, requires identifying an IV that satisfies the orthogonality condition (i.e., a variable that is strongly correlated with the level of market participation but not directly correlated with our outcome variables). Following Rao and Qaim (2011) and Rao, Brümmer, and Qaim (2012), we used the availability of public transportation in the village as an IV or exclusive restriction. Public transportation is likely to increase market participation, and this is plausible because in most cases, farmers must deliver their products themselves to the cowpea market. We therefore included the availability of public transportation in Z*i*.To test whether the instrument is relevant, we used the Anderson canonical correlations test (Baum, Schaffer, & Stillman, 2007).

In addition to the IV approach outlined above, we also estimated the dose–response functions (DRFs) and their derivatives (marginal treatment effects) following Cerulli (2015). Unlike previous DRFs models, for example, Bia and Mattei (2008) which require the normality assumption to be satisfied, this model does not need the full normality assumption, and it is well‐suited when many individuals have a treatment level of zero (Cerulli, 2015). For the sake of brevity, we do not present the estimation niceties of the DRF model, but Cerulli (2015) gives an overt description of the model.

To test whether selling to rural or urban traders influences HDD, food expenditure, and household income, we used the control function approach (Rivers & Vuong, 1988; Wooldridge, 2015). Unlike the cowpea market participation indicator, which was a continuous variable, the decisions of whether to sell to rural or urban traders are dummy variables; hence, we used the appropriate modeling procedure that considers the binary nature of our indicator variables. We can redefine Equation 2 as

(3) $$T_{i}=1\left[\alpha_{0}+\alpha_{1}Z_{i}+\varepsilon_{i}\geq 0\right]$$

where *Z_i_* is a vector of the determinants of the decision to sell to rural traders and urban traders; 1 [.] is an indicator function whose value is 1 if the statement inside the brackets is true, and 0 otherwise. Note that in this study, rural traders are defined as those traders who buy cowpea within the farmers' village/markets. Urban traders on the other hand are defined as traders who buy cowpea directly from farmers and rural traders in the main district markets. Denoting the observed HDD, food expenditure, and household income of the participants in the rural or urban market and nonparticipants by *Y_i_*_0_ and *Y_i_*_1_, then we can specify the potential outcomes as

(4) $$Y_{i0}=\alpha_{i0}+X\beta_{i0}+\mu_{i0}$$

(5) $$Y_{i1}=\alpha_{i1}+X\beta_{i1}+\mu_{i1}$$

where *X_i_* is defined as above (Equation 1), *β* represent the parameters to be estimated, and *μ_i_*_0_ is unobserved random component. The observed treatment and outcome can be expressed as

(6) $$Y_{i}=T_{i}Y_{i1}+\left(1-T_{i}\right)Y_{i0}$$

*T_i_* in Equation 6 is also potentially endogenous and this may arise because of unobserved heterogeneity, reverse causality, or measurement error, leading to biased estimates (Ogutu et al., 2019; Ogutu & Qaim, 2019). That is, the treatment variable *T_i_* may be correlated with the error terms in Equations 4 and 5. To break the correlation between the possibly endogenous treatment variable and unobservables affecting the outcome variable, we used the IV control function approach (CFA). In addition to the instrument mentioned above, we included ownership of donkey/ox cart in the rural trader model as an additional exclusion restriction. Donkey/ox carts are importantin northern Nigeria where livestock rearing is an integral part of the farming system. Since they are usually used in the transportation of crop produce to the market, we envisage that this variable is correlated with our treatment variable but not directly with the outcome variables.

The estimation of the CFA proceeds in two steps. In the first step, we estimated a probit model of the choice to sell cowpea to rural or urban traders (Equation 3, including the instruments) and obtained the generalized residuals. The predicted residuals were then included as additional covariates in the second‐stage regression of the outcome variables (Equation 6). Since the outcome variables—HDD, food expenditure, and household income—are all continuous variables, we estimated the outcome equations using ordinary least squares regression (OLS). The final estimate that we get is the average treatment effect on the treated ATT, that is, the effect for only those households who sold their marketable surplus to rural and urban traders.

### 2.2 Data collection

The data for this study came from a nationally representative sample survey of 1,525 cowpea producing households conducted in 2017 by the International Institute of Tropical Agriculture (IITA) under the Tropical Legumes III project. A survey questionnaire was designed using computer‐assisted personal interviewing (CAPI) based software called Surveybe and administered by trained enumerators who collected data from households through personal interviews. The survey targeted 10 states—Borno, Bauchi, Gombe, Jigawa, Kaduna, Kano, Katsina, Kebbi, Sokoto, and Zamfara—which represent about 75% of the total cowpea production in Nigeria. These states mainly fall within the Sudan Savanna, which is the major agro‐ecological zone for cowpea production in Nigeria. A multistage stratified sampling procedure was used to select the households. In the first stage, the 10 states were grouped into two geopolitical regions—three states in northeast and seven states in northwest. Only three states were considered (Borno, Bauchi, and Gombe) from the northeast region due to the security problems experienced in other states in the region during the survey. A list of villages and Local Government Areas (LGAs) located in the 10 states of the two regions was obtained from the National Population Commission (NPC).

In the second stage, 25 and 13 LGAs were selected in each region using probability proportional to size (PPS) sampling. In the third stage, five cowpea producing villages were then randomly selected from each of the selected LGAs. Following the selection of the villages, a sampling frame was developed for cowpea‐growing households in the selected villages with the help of the extension agents from the Agricultural Development Programs (ADPs). In the final stage, eight households were randomly selected from each selected village resulting in a total sample of 1,525 households (995 households in the northwest region and 530 households in the northeast region).^4^

The survey collected valuable information on the socioeconomic characteristics of the sample households, quantity of cowpea sold, marketing costs, and type of traders to whom farmers sell their produce.

## 3 RESULTS AND DISCUSSION

### 3.1 Descriptive statistics

Table 1 shows the definitions and descriptive statistics of the key variables in our study. Our market participation variable is represented by quantity of cowpea sold. Results indicate that on average households sold about 633 kg of cowpea to various players in the market with about 48% of them selling to rural traders and 15% to urban wholesalers. Considering that the average production in our sample was 927 kg, the results suggest that over 60% of the households sold their cowpea. The proportion of cowpea sold was much higher than that reported by Gondwe et al. (2017) in Zambia and Mignouna et al. (2016) in Nigeria.

**TABLE 1:** Definitions and summary statistics of the variables used in the analysis

Variables	Mean	*SD*	Min	Max
Treatment variables				
Quantity of cowpea sold (kg)	632.55	762.14	0	4,500
Proportion of households who sold to rural traders	0.48	0.500	0	1
Proportion of households who sold to urban traders	0.15	0.36	0	1
Outcome variables				
Household dietary diversity (HDD) (i.e., number of food groups consumed by HH in the past 1 week)	8.67	1.23	0	12
Food expenditure per adult equivalent (Naira)	78,425	45,916	8,921.538	525,341.30
Total household income per capita (Naira)	116,546	112,571	5,341	1.309e+06
Independent variables				
Age of the household head (years)	44.14	12.09	18	95
Sex of the household head (1 = Male)	0.96	0.19	0	1
Education of the household head (1 = HH head completed 9 years of education)	0.04	0.18	0	1
Number of adults (15–59 years) in the household	7.36	2.42	1	16
Livestock ownership in Tropical Livestock Units (TLU)	3.11	4.36	0	46
Land owned (ha)	4.73	5.16	0.500	110
Access to off‐farm income (1 = HH has access to off‐farm income)	0.85	0.36	0	1
Credit constrained (1 = HH is credit constrained)	0.32	0.47	0	1
Average one‐way transport cost to the main market (Naira)	159.90	112.0	0	1,000
Radio ownership (1 = HH owns radio)	0.57	0.50	0	1
Number of contacts with extension agents	1.92	5.02	0	40
Friends or relatives in leadership positions in formal or informal institutions (1 = HH has friends/relatives in leadership position)	0.37	0.48	0	1
Kinship (number of relatives within and outside village household can rely upon for critical support)	12.69	11.80	0	125
Number of years the head of the household has been living in this village	36.78	14.16	1	85
Member/s of formal and informal groups/institutions (1 = HH is a member of a group)	0.39	0.49	0	1
Adoption of improved cowpea varieties (1 = HH adopted improved cowpea varieties)	0.42	0.49	0	1
Wealth index	−0.03	1.89	−2.82	11.07
Distance from residence to field (minutes)	26.221	28.035	1	480
Distance to the local (village) market from residence (minutes)	42.54	61.54	0	99
Instrumental variables				
Presence of public transport (1 = yes)	0.62	0.49	0	1
Owns donkey/ox cart (1 = yes)	0.16	0.37	0	1

The outcome variables used in the study are the HDD and food expenditure which are proxies for food security , and the total household income , a proxy for the welfare of the farm households. The household HDD was constructed following the guidelines provided by Kennedy, Ballard, and Dop (2010). During the survey, households were asked to mention the food items they consumed in the past 7 days ranging from cereals, vegetables, proteins to beverages and condiments. These food items were classified into 12 food groups, each with a score of 1. According to Kennedy et al. (2010), the HDD is a qualitative measure of food consumption that reflects household access to a variety of foods and is also a proxy for nutrient adequacy of the diet of individuals. It is also meant to reflect the economic ability of a household to access a variety of foods. Table 1 shows that the HDD ranged from 0 to 12 with a mean value of about 9. This implies that on average, most of the households had reasonably good access to a variety of foods. Food expenditure is an important measure of food security as it is an indicator of economic vulnerability, that is, it approximates the losses experienced when food prices rise (Lele, Masters, Kinabo, & Meenakshi, 2016; Moltedo, Troubat, Lokshin, & Sajaia, 2014; Smith & Subandoro, 2007). Food expenditure includes the total food purchased by the household, the consumption of food produced by the household, and any food received by the household either through aid or in kind. The results show that on average, households spent ₦78,425 on food purchases, accounting for two‐thirds of the household income. The average household income was about ₦116,546. Household income which is an indicator of farmers' well‐being includes income from crops, livestock and livestock products, and off‐farm income (e.g., salaries, remittances, farm labor wage income, pension income, and income from business).

Table 1 further presents the household characteristics such as age, sex, education, cultivated land, number of adults in the household, and access to off‐farm income. About 96% of the sample households were male headed, with about 4% of the households attending junior secondary school education. The number of adult members of the household between the ages of 15 and 59 is used as a proxy for household labor endowment. On average, each household had about seven adult members. Almost 85% of the sample households had access to off‐farm income. Off‐farm income is an indication of the dependence on off‐farm employment in the household's community and among neighboring communities and may affect the individual household's labor allocation and cash earnings (Smale & Mason, 2014). Kinship, number of years the head of the household has been living in this village, friends or relatives in leadership positions in formal or informal institutions and member of formal and informal groups/institutions are all important indicators of social capital. About 59% of the households owned a radio. Radio ownership is important especially for the decision to participate in markets because radio ownership can facilitate access to production, market and price information (Olwande et al., 2015). Following Aguilar, Carranza, Goldstein, Kilic, & Oseni, (2015), we constructed a wealth index using principal component analysis (PCA) in which we considered all the assets owned by the household such bicycles, motorbikes cars, and television sets. Households with more assets are expected to participate in the market more fully than those with fewer assets. Previous studies have shown that improved cowpea varieties out yield local varieties, implying that a household's marketable surplus can be influenced by productivity (Kamara et al., 2010; Olwande et al., 2015). To capture this, we included an improved cowpea adoption dummy, which shows that about 42% of the households planted improved cowpea varieties in the 2016 cropping season. In participating in the input and output markets, households also incur transaction costs (Alene et al., 2008; Barrett, 2008; Olwande et al., 2015). The average one‐way transport cost to the main market, distance to the village market, and distance from the homestead to farm are proxies for transaction costs incurred by farmers in transporting their produce to the market. It takes an average of 43 min for farmers to transport produce to the nearest village markets. Lastly, about 62% of the households had access to public transport and about 15% owned donkey/ox carts.

Table 2 shows the outcome variables disaggregated by cowpea market participation. Note in Table 2, market participation is defined by a discrete choice variable and not continuous variable. The results indicate that cowpea market participants had higher food expenditures and incomes as compared to their nonparticipants. Participants spent about ₦11,736 more on food purchases than nonparticipants. Similarly, cowpea market participants had on average ₦18,867 more income than nonparticipants. While these results may suggest that cowpea market participation can be beneficial to the households, it will be misleading to conclude that market participation had impact on food expenditure and household income since descriptive analysis does not control for both observed and unobserved characteristics. To effectively assess its impact, we turn to multivariate analysis presented in the subsequent sections.

**TABLE 2:** Outcome variables by cowpea market participation

Variable	Participant in cowpea market	Nonparticipant	Difference
Household dietary diversity	8.68	8.65	0.02
Food expenditure (Naira)	79,499.57	67,763.16	11,736.41**
Total household income (Naira)	118,116.70	99,249.45	18,867.30*

Note

The difference is measured by the two‐sample *t* test with equal variances.

* *p* < .10.

** *p* < .05.

### 3.2 Impact of cowpea market participation on household dietary diversity, food expenditure, and household income

We estimated the impact of cowpea market participation on HDD, food expenditure, and household income considering both observed and unobserved characteristics using an instrumental variable regression outlined in Section 2. But first, we estimated the determinants of cowpea market participation using ordinary least squares (OLS), and the results are presented in Table A1, ^5^ in the appendix. Availability of public transport in the village is the instrumental variable used in the estimation. The availability of public transport in the village is expected to influence market participation without directly affecting our impact variables, making it a good candidate to be used as instrument. Results in Table A1 show that the availability of public transport in the villages is an important determinant of level of participation in the cowpea market, suggesting that it can be a relevant instrument to identify our IV model. The Anderson canonical correlations likelihood‐ratio test of whether the equation is identified, that is, that the excluded instruments are relevant is reported in Table 3 and shows a rejection of the null hypothesis, suggesting that the model is identified and that the instrument is relevant.

**TABLE 3:** Effect of cowpea market participation on household dietary diversity, food expenditure, and household income (instrumental variable regression)

Variable	Household dietary diversity	Ln (Food expenditure )	Ln (Total household income)
Quantity of cowpea sold	0.00** (0.00)	0.00** (0.00)	0.00* (0.00)
Age of the household head	0.00 (0.01)	−0.01* (0.00)	−0.01*** (0.00)
Sex of the household head	−0.05 (0.30)	0.00 (0.17)	0.23** (0.10)
Education	−0.17 (0.27)	−0.07 (0.15)	−0.04 (0.10)
Number of adults in the household	−0.03 (0.02)	−0.08*** (0.01)	−0.08*** (0.01)
Livestock ownership	−0.06* (0.03)	−0.02 (0.02)	0.01 (0.01)
Total land owned	−0.12** (0.05)	−0.07** (0.03)	−0.04* (0.02)
Access to off‐farm income	0.10 (0.20)	−0.11 (0.11)	0.37*** (0.08)
Credit constrained	0.08 (0.11)	−0.08 (0.06)	−0.16*** (0.05)
Average one‐way transport cost (per person) to the main market	−0.00 (0.00)	−0.00 (0.00)	−0.00 (0.00)
Number of contacts with extension agents	−0.02 (0.01)	−0.01 (0.01)	−0.01 (0.00)
Radio ownership	−0.12 (0.11)	−0.02 (0.06)	−0.06 (0.04)
Friends or relatives in leadership positions in formal or informal institutions	−0.14 (0.14)	−0.07 (0.08)	0.11** (0.05)
Kinship	−0.01** (0.01)	−0.00 (0.00)	−0.00 (0.00)
Number of years the head of the household has been living in this village	0.01 (0.01)	0.01* (0.00)	0.00 (0.00)
Memberof formal and informal groups/institutions	0.25** (0.12)	0.15** (0.07)	0.02 (0.05)
Adoption of improved cowpea varieties	0.12 (0.13)	0.04 (0.08)	0.15*** (0.05)
Wealth index	0.12** (0.05)	0.03 (0.03)	0.07*** (0.02)
Distance from residence to field	−0.01* (0.00)	−0.00* (0.00)	−0.00 (0.00)
Distance to the local (village) market from residence	−0.00 (0.00)	−0.00* (0.00)	0.00 (0.00)
Northwest dummy	0.25 (0.22)	0.20 (0.13)	0.12 (0.08)
Constant	8.22*** (0.40)	11.36*** (0.24)	11.45*** (0.16)
Anderson canon correlation statistic (identification/IV relevance test)	*χ* ^2^ (1) = 9.82; *p* > *χ* ^2^ = .07		
Observations	1,513	1,513	1,513

Note

Village cluster robust standard errors in parentheses.

* *p* < .10.

** *p* < .05.

*** *p* < .001

Table 3 shows the results from our IV model from Equation 1. Specifically, the first row of Table 3 shows the impact of quantity of cowpea sold on HDD, food expenditure, and household income. The results indicate that cowpea market participation significantly increasd HDD, food expenditure, and household income. A 10% increase in the quantity of cowpea sold increases the food expenditure and income by 1.6% and 0.7%, respectively. These results are consistent with the results found by Montalbano et al. (2018) and Muriithi and Matz (2015) who found that maize and vegetable market participation increased food consumption, nutrition and total household income in Uganda and Kenya, respectively.

**FIGURE 1:**
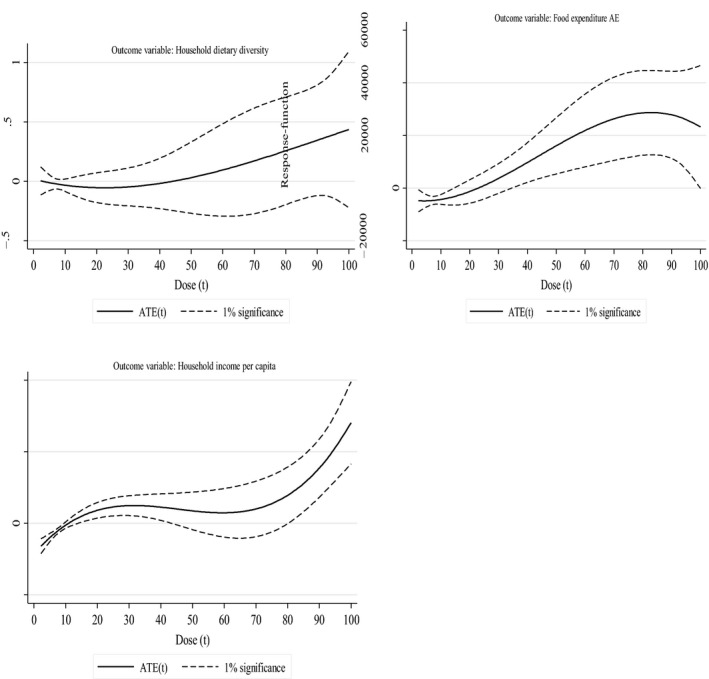
Estimated dose–response functions (average treatment effect) of cowpea market participation on household dietary diversity, food expenditure , and household income

To evaluate the effect of cowpea market participation over the entire sample of the distribution, we also estimated the dose–response functions. The dose–response functions in a way can also be viewed as a robustness check for the IV results since the functions are estimated based on observed characteristics and ignore the unobserved characteristics. Coupled with the DRFs, we also estimated the derivatives of the DRFs, that is, the marginal treatment effects to capture the heterogeneity in treatment effects. Figure 1 shows the estimated dose–response functions (average treatment effect) of cowpea market participation on HDD, food expenditure , and household income . Each estimated DRF is accompanied by 1% confidence bands. The *x*‐axis shows the dose, which is the quantity of cowpea sold, scaled to be between 0 and 100 while the *y*‐axis measures the average treatment effect (ATE). The results show that as the amount of cowpea sold increases, so do the HDD, food expenditure, and household income. The results show that food expenditure increases from below zero to a maximum of around ₦25,000 with an increase in the quantity of cowpea sold. Likewise, household income increases, reaching a maximum of about ₦140,000 with an increase in the amount of cowpea sold. These results are quite consistent with the IV results presented in Table 3.

**FIGURE A1:**
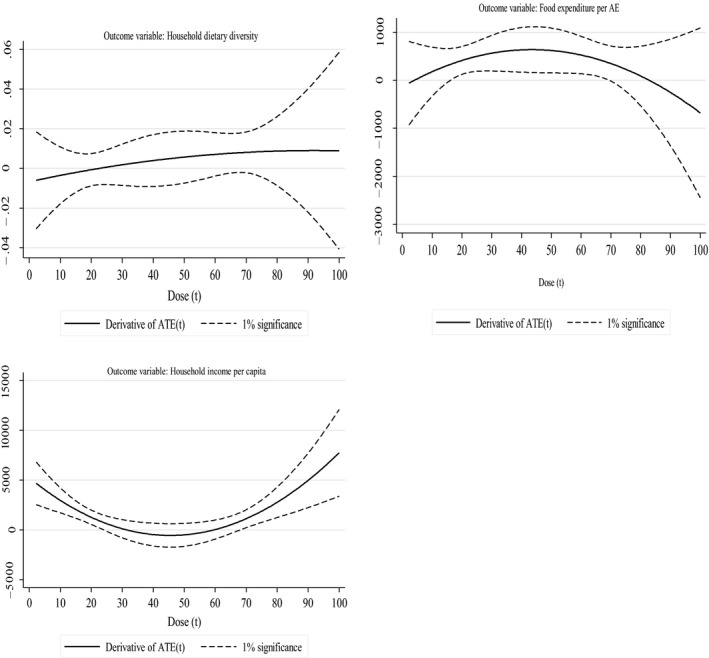
Derivative of the dose–response functions (marginal treatment effects) of market participation on household dietary diversity, food expenditure , and household income

The marginal treatment effects graphs (Figure A1 in the appendix) show that the returns to cowpea market participation are heterogeneous, and generally, the conclusions are comparable to the DRFs. The slopes of all the MTE curves are positive, indicating positive selection bias, that is, cowpea market participation is most effective in increasing HDD, food expenditure, and household income for farmers who sold a larger amount of cowpea. This is consistent with the results found by Manda, Khonje, Alene, and Gondwe (2017) for groundnuts in Zambia and Wossen et al. (2018) for cassava in Nigeria.

### 3.3 Impact of cowpea market choice on household dietary diversity, food expenditure, and household income

To fully understand the benefits of participating in the cowpea market, we analyzed the effect of the farmers' choice of either selling to rural traders or urban wholesalers on the same outcome variables as above. We estimated an instrumented control function model as outlined in Section 2. We used the availability of public transport as an instrument to identify the urban trader choice model. To identify the rural trader choice model, we included the variable, ownership of donkey/ox carts in addition to the availability of public transport variable for reasons explained above. Tables A2 and A3 in the appendix present the results of the endogeneity tests for the treatment variables. We performed a Wald test to determine whether the estimated correlations between the treatment‐assignment and potential‐outcome models were different from zero. The null hypothesis is that the correlations are jointly zero and rejection of the null hypothesis suggests endogeneity. Results in Table A3 show that we can reject the null hypothesis of no endogeneity at 10% and 5% for the HDD, food expenditure, and income equations, implying that without controlling for this endogeneity, our results would be biased. This provides the justification for using the instrumented control function approach.

Even though our main objective was to assess the benefits of either choice of the market, we discuss briefly the determinants of the choice of whether to sell to rural or urban traders in Table 4 (first stage control function results).^6^ Results in Table 4 show that the determinants of the decision of whether to participate in the rural or urban markets are different in terms of both magnitude and direction. For instance, participation in urban markets increases with the age of the household head. Livestock ownership increases the participation in rural markets but reduces participation in the urban markets. This is probably because livestock is usually used to transport produce to village markets where in most cases the roads are in bad conditions and public transportation is not available. On the other hand, in urban areas, transportation of produce to the market can easily be done using public transport because generally the roads are in better conditions than those in the rural areas. Likewise, access to off‐farm income has a positive effect on the decision to sell to rural traders and has a negative effect on urban market participation. Generally, the social capital variables had statistically significant effects on the decision to sell to rural traders while insignificant for the urban traders. The distance to the nearest market had a positive effect on the decision to sell to rural traders and negative on the urban traders. This is probably because of high transaction costs associated with transporting the marketable surplus to the market. Most of the village markets are near farmers' residences while those in urban markets are quite distant from the farmers' residences. Previous studies (e.g., Alene et al., 2008; Barrett, 2008; Renkow, Hallstrom, & Karanja, 2004) have shown that transaction costs (e.g., distance to the nearest market) prevent some of the farmers from participating in the market and in our case, prevents farmers from accessing urban markets offered by the urban wholesalers. Finally, our instrumental variables were statistically significant in explaining the decision to sell to the rural and urban traders.

**TABLE 4:** Determinants of the cowpea market choice

Variable	Rural traders	Urban traders
Age of the household head	−0.00 (0.00)	−0.00 (0.00)
Sex of the household head	−0.17 (0.17)	0.41* (0.23)
Education	−0.21 (0.18)	0.29 (0.21)
Number of adults in the household	0.01 (0.02)	0.01 (0.02)
Livestock ownership	0.04*** (0.01)	−0.03** (0.01)
Land owned	0.02** (0.01)	−0.01 (0.01)
Access to off‐farm income	0.34*** (0.10)	−0.46*** (0.11)
Credit constrained	0.13* (0.07)	−0.03 (0.09)
Average one‐way transport cost (per person) to the main market	−0.00 (0.00)	0.00** (0.00)
Number of contacts	0.02** (0.01)	−0.01 (0.01)
Radio ownership	−0.27*** (0.07)	0.12 (0.09)
Friends or relatives in leadership positions in formal or informal institutions	0.15* (0.08)	−0.09 (0.10)
Kinship	0.01** (0.00)	−0.01 (0.00)
Number of years the head of the household has been living in this village	−0.00 (0.00)	0.00 (0.00)
Member of formal and informal groups/institutions	−0.30*** (0.08)	−0.13 (0.10)
Adoption of improved cowpea varieties	−0.06 (0.07)	−0.30*** (0.09)
Wealth index	0.03 (0.02)	0.08** (0.02)
Distance from residence to field	0.00 (0.00)	−0.00** (0.00)
Distance to the local (village) market from residence	0.00** (0.00)	−0.00** (0.00)
Northwest dummy	0.06 (0.07)	−0.25** (0.09)
Presence of public transport	0.17** (0.07)	0.39*** (0.09)
Owns donkey/ox cart	0.20** (0.09)	
Constant	−0.28 (0.24)	−0.84** (0.30)
Observations	1,513	1,513

Note

Robust standard errors in parentheses.

* *p* < .10.

** *p* < .05.

*** *p* < .001.

After controlling for observed and unobserved characteristics, the impact of the choice of whom to sell cowpea (i.e., either to rural or urban traders) from the control function model is presented in Table 5 below. The results show that selling to rural traders increases HDD by 38%, food expenditure and household income by 17%, each. Similarly, selling to urban traders increased HDD by 55%, food expenditure by 16%, and household income increased by 13%. The magnitudes of the percentage increase were not very different between the two market outlets. These results are consistent with other studies (e.g., Asfaw, Lipper, Dalton, & Audi, 2012; Montalbano et al., 2018; Sibhatu et al., 2015; Stifel & Minten, 2017) which indicate that market access/participation increases the dietary diversity and well‐being of farm households. There are two major pathways through which market participation can affect dietary diversity. One pathway is through an increase in agricultural production, followed by more marketable surplus and income. Market participation leads to an increase in agricultural production which in turn leads to more marketable surplus (Stifel & Minten, 2017). The market surplus increases income for farmers which consequently increase their ability to buy more diverse foods from the market (Sibhatu et al., 2015). The other pathway is through transaction cost reduction followed by more marketable surplus and income. That is, participation in the local markets provided by rural traders reduces the transaction costs associated with the marketing of cowpea produce, which in turn leads to more marketable surplus and more income for farmers which consequently increase their ability to buy more diverse foods from the market (Sibhatu et al., 2015). On the flip side, lack of local market access increases transaction costs and leads to less marketable agricultural surplus, thus resulting in less food and fewer food items purchased (Stifel & Minten, 2017).

**TABLE 5:** Impact of market choice on household dietary diversity, food expenditure, and household income

Outcome variable	Buyer type	Participant in market type	Nonparticipant	ATT	Percent increase
Household dietary diversity	Rural traders	8.70	6.31	2.39* (1.23)	38
Ln (Food expenditure in adult equivalent)	Rural traders	11.13	9.55	1.58** (0.70)	17
Ln (Total household income)	Rural traders	11.41	9.73	1.68** (0 0.83)	17
Household dietary diversity	Urban traders	8.64	5.57	3.07*** (0.93)	55
Ln (Food expenditure in adult equivalent)	Urban Traders	11.10	9.59	1.51** (0.48)	16
Ln (Total household income)	Urban traders	11.32	10.05	1.27** (0.53)	13

Note

Robust standard errors in parentheses.

* *p* < .10.

** *p* < .05.

*** *p* < .001.

## 4 CONCLUSIONS AND POLICY RECOMMENDATIONS

This study estimated the impact of cowpea market participation on household dietary diversity, food expenditure per adult equivalent, and household income per capita using data from a nationally representative sample of over 1,500 farm households in northern Nigeria. We used a combination of instrumental variable techniques and dose–response functions to achieve our objective.

Using the quantity of cowpea sold as an indicator of cowpea market participation, we found that on average farmers sold about 633 kg of cowpea to various cowpea traders. The results from our study also showed that, ceteris paribus, cowpea market participation significantly increased household dietary diversity, food expenditure, and household income, consistent with other studies on market participation and commercialization of agricultural produce. Cowpea market participation increased food expenditure by 1.6% and household income by 0.7% with a 10% increase in the amount of cowpea sold. The results suggest that the contribution of cowpea market participation to household income led to higher food expenditures and a more diversified diet. The effects on income and expenditure are plausible and consistent with other studies, considering that we only looked at cowpea. Ogutu et al. (2019) for instance examined the impact of agricultural commercialization (including crops and livestock), on income and poverty in Kenya. They found that commercialization increased income by 17% and reduced the prevalence of poverty by 5.1 percentage points. Similar results were obtained by Radchenko and Corral (2018) on food security in Malawi and Seng (2016) in Cambodia. Unlike other studies which have not explored the benefits of different markets channels of smallholder market participation on household food security and income, we showed in this study that selling cowpea to rural traders on average increased food security as measured by the household dietary diversity scores by 38% while selling to urban traders increased food security by 55%. Specifically, the results suggest that farmers who sold to urban traders had more diversified diets than those who sold to rural traders. In urban areas, there is usually variety of foods that are available for purchase as compared to rural areas where there is a limited choice; hence, the likelihood of households who had to travel to urban areas to sell their cowpea of having a diversified diet is higher than those who sold within the rural areas. Contrary to the common view that rural traders exploit small‐scale farmers, results show that selling cowpea to rural traders on average increased household income by 17% while selling cowpea to urban traders increased by 13%. Rural traders are economic agents performing a function for which they are being remunerated, and at the same time, this function provides benefits to farmers as shown by our results. The results from this study show that these traders can be one of the solution to the seemingly nonexistent structured grain markets in rural areas. In most parts of the rural northern Nigeria, the road infrastructure is not in a good condition, making it very difficult for farmers to access distant urban markets. It also makes it very difficult for farmers to access market information from extension agents. Rural traders fill in these gaps by providing local stable market‐outlet services, lowered search and transport costs and through interlinked contracts and may provide credit, inputs, and information (Kassie, Jaleta, Shiferaw, Mmbando, & Mekuria, 2013; Sitko & Jayne, 2014). It is therefore important for the government to recognize that rural traders also play an important role in the marketing of agricultural produce and to create an enabling environment in which commodity traders are encouraged to participate in the cowpea market. Precisely, the provision of credit facilities would greatly encourage these traders to buy produce even from hard to reach areas where most of the big traders are not present. Second, availability of public transportation is important in encouraging farmers to access urban markets; hence, public infrastructure investments in the form of feeder road construction and maintenance in the distant villages are encouraged, which in the long run can translate into improved cowpea productivity and welfare of smallholder farmers in not only Nigeria, but sub‐Saharan Africa as a whole where market access is particularly a problem.

## ENDNOTES

^1^ This is based on the last official estimate of 2009–2010.

^2^ See a Lowenberg‐DeBoer and Germaine (2008) and Mishili et al. (2009) for a detailed description and analysis of the cowpea value in Nigeria.

^3^ The HDDS are used to measure HDD.

^4^ A total of 1,530 households were interviewed but five households were dropped because the data were incomplete.

^5^ For the sake of brevity, we do not discuss these results.

^6^ The second stage results are presented in Tables A2 and A3 in the appendix.

## APPENDIX 1

**TABLE A1:** Determinants of cowpea market participation

Variable	Quantity of cowpea sold	Ln (Quantity of cowpea sold)
Age of the household head	−0.71 (2.10)	−0.01** (0.01)
Sex of the household head	50.23 (90.64)	0.02 (0.22)
Education	67.34 (87.34)	0.00 (0.26)
Number of adults in the household	4.91 (9.18)	0.01 (0.02)
Livestock ownership	25.24*** (6.13)	0.03** (0.01)
Total land owned	48.76*** (11.16)	0.08*** (0.02)
Access to off‐farm income	133.97** (47.82)	0.17 (0.12)
Credit constrained	8.17 (38.71)	−0.06 (0.11)
Average one‐way transport cost (per person) to the main market	0.71** (0.27)	0.00* (0.00)
Number of contacts with extension agents	5.76 (4.74)	0.01* (0.01)
Radio ownership	32.08 (33.20)	0.11 (0.10)
Friends or relatives in leadership positions in formal/informal institutions	45.13 (42.66)	0.16 (0.11)
Kinship	3.04 (2.14)	0.01** (0.00)
Number of years the head of the household has been living in this village	−2.89 (2.10)	−0.00 (0.00)
Member of formal and informal groups/institutions	−31.74 (41.20)	−0.24** (0.11)
Adoption of improved cowpea varieties	−31.92 (39.80)	−0.08 (0.11)
Wealth index	15.48 (12.68)	−0.03 (0.03)
Distance from residence to field	2.43** (0.78)	0.00 (0.00)
Distance to the local (village) market from residence	0.24 (0.40)	0.00 (0.00)
Northwest dummy	−148.41** (52.61)	−0.56*** (0.12)
Presence of public transport	113.43** (40.84)	1.18*** (0.12)
Constant	44.84 (124.07)	4.89*** (0.32)
Observations	1,513	1,513

Note

Village cluster robust standard errors in parentheses

* *p* < .10.

** *p* < .05.

*** *p* < .001.

**TABLE A2:** Determinants of household dietary diversity, food expenditure, and household income (rural traders)

Variable	Household dietary diversity		Ln (Food expenditure )		Ln (Total household income)	
	Nonrural traders	Rural traders	Nonrural traders	Rural traders	Nonrural traders	Rural traders
Age of the household head	−0.00 (0.01)	0.01** (0.01)	−0.01** (0.00)	−0.00 (0.00)	−0.01* (0.00)	−0.01* (0.00)
Gender of the household head	0.23 (0.25)	0.25 (0.29)	0.17 (0.15)	0.15 (0.13)	0.49** (0.18)	0.17 (0.12)
Education	0.35 (0.27)	−0.09 (0.32)	0.09 (0.14)	0.28* (0.15)	0.26 (0.16)	−0.19 (0.17)
Number of adults in the household	−0.04* (0.02)	−0.01 (0.02)	−0.08*** (0.01)	−0.09*** (0.01)	−0.10*** (0.02)	−0.08*** (0.01)
Livestock ownership	−0.01 (0.02)	−0.04* (0.02)	0.00 (0.01)	0.00 (0.01)	0.03** (0.01)	0.01 (0.01)
Total land owned	−0.03* (0.01)	−0.02 (0.01)	−0.01 (0.01)	−0.01 (0.01)	−0.02* (0.01)	−0.00 (0.01)
Access to off‐farm income	0.21 (0.20)	−0.17 (0.25)	−0.05 (0.15)	−0.19 (0.12)	0.19 (0.14)	0.44*** (0.13)
Credit constrained	−0.02 (0.12)	0.02 (0.12)	−0.17** (0.07)	−0.07 (0.06)	−0.25** (0.08)	−0.13** (0.06)
Average one‐way transport cost (per person) to the main market	0.00 (0.00)	0.00 (0.00)	0.00* (0.00)	0.00** (0.00)	0.00* (0.00)	0.00 (0.00)
Number of contacts	−0.02 (0.02)	−0.03* (0.02)	−0.01 (0.01)	−0.00 (0.01)	−0.01 (0.01)	−0.01* (0.01)
Radio ownership	0.37** (0.16)	−0.09 (0.19)	0.24** (0.10)	0.09 (0.08)	0.17 (0.11)	−0.01 (0.09)
Friends or relatives in leadership positions in formal or informal institutions	−0.32** (0.14)	0.11 (0.15)	−0.03 (0.08)	−0.11* (0.07)	0.01 (0.09)	0.17** (0.08)
Kinship	−0.02*** (0.01)	−0.01 (0.01)	−0.00 (0.00)	0.00 (0.00)	−0.00 (0.00)	−0.00 (0.00)
Number of years the head of the household has been living in this village	0.01** (0.01)	−0.01** (0.01)	0.01* (0.00)	0.00 (0.00)	0.00 (0.00)	−0.00 (0.00)
Member of formal and informal groups/institutions	0.69*** (0.18)	0.13 (0.20)	0.38*** (0.10)	0.14 (0.09)	0.24** (0.12)	−0.00 (0.10)
Adoption of improved cowpea varieties	0.15 (0.11)	0.02 (0.11)	0.04 (0.07)	−0.01 (0.05)	0.19** (0.07)	0.12** (0.06)
Wealth index	0.16*** (0.03)	0.12*** (0.04)	0.06** (0.02)	0.04** (0.02)	0.08*** (0.02)	0.07*** (0.02)
Distance from residence to field	−0.00 (0.00)	0.00 (0.00)	−0.00 (0.00)	0.00 (0.00)	0.00 (0.00)	0.00 (0.00)
Distance to the local (village) market from residence	−0.00 (0.00)	−0.00 (0.00)	−0.00** (0.00)	−0.00** (0.00)	−0.00 (0.00)	0.00 (0.00)
Northwest dummy	−0.15 (0.11)	−0.22* (0.11)	−0.12* (0.06)	−0.02 (0.05)	−0.11 (0.07)	0.08 (0.06)
Constant	7.32*** (0.61)	9.98*** (0.90)	10.81*** (0.38)	12.38*** (0.42)	10.68*** (0.43)	11.98*** (0.41)
Observations	732	785	732	785	732	785
Wald test for endogeneity of treatment variable	*χ* ^2^ (2) = 5.37; *p* > *χ* ^2^ = .07		*χ* ^2^ (2) = 5.30; *p* > *χ* ^2^ = .07		*χ* ^2^ (2) = 4.51; *p* > *χ* ^2^ = .10	

Note

Robust standard errors in parentheses.

* *p* < .10.

** *p* < .05.

*** *p* < .001.

**TABLE A3:** Determinants of household dietary diversity, food expenditure, and household income (urban traders)

Variable	Household dietary diversity		Ln (Food expenditure )		Ln (Total household income)	
	Nonurban traders	Urban traders	Nonurban traders	Urban traders	Nonurban traders	Urban traders
Age of the household head	0.00 (0.01)	0.00 (0.01)	−0.01** (0.00)	−0.01** (0.00)	−0.01*** (0.00)	−0.01* (0.01)
Sex of the household head	−0.12 (0.22)	−0.41 (0.36)	−0.03 (0.11)	0.01 (0.17)	0.21* (0.12)	−0.27 (0.39)
Education	−0.33 (0.26)	0.33 (0.31)	−0.03 (0.12)	−0.01 (0.15)	−0.08 (0.13)	−0.02 (0.20)
Number of adults in the household	−0.01 (0.02)	−0.04 (0.03)	−0.08*** (0.01)	−0.06*** (0.01)	−0.08*** (0.01)	−0.08*** (0.02)
Livestock ownership	0.02 (0.01)	0.00 (0.03)	0.03*** (0.00)	0.01 (0.01)	0.04*** (0.01)	0.04** (0.02)
Total land owned	0.01 (0.01)	−0.02 (0.01)	0.01 (0.00)	0.00 (0.01)	0.00 (0.00)	0.01 (0.01)
Access to off‐farm income	0.83*** (0.15)	0.01 (0.24)	0.29** (0.12)	−0.11 (0.10)	0.57*** (0.09)	0.67*** (0.17)
Credit constrained	0.02 (0.10)	0.52*** (0.15)	−0.08* (0.04)	−0.03 (0.06)	−0.12** (0.05)	−0.34** (0.11)
Average one‐way transport cost (per person) to the main market	−0.00 (0.00)	−0.00 (0.00)	0.00 (0.00)	−0.00 (0.00)	0.00 (0.00)	0.00 (0.00)
Number of contacts	0.00 (0.01)	−0.02 (0.01)	0.01** (0.00)	−0.00 (0.01)	0.00 (0.00)	−0.01 (0.01)
Radio ownership	−0.15 (0.10)	−0.09 (0.15)	−0.01 (0.05)	0.03 (0.06)	−0.06 (0.05)	−0.13 (0.11)
Friends or relatives in leadership positions in formal or informal institutions	0.08 (0.11)	−0.15 (0.16)	0.02 (0.05)	0.09 (0.06)	019*** (0.05)	0.05 (0.11)
Kinship	−0.00 (0.00)	−0.00 (0.01)	0.00** (0.00)	−0.00 (0.00)	0.00 (0.00)	0.00 (0.00)
Number of years the head of the household has been living in this village	−0.00 (0.00)	0.00 (0.01)	0.00 (0.00)	0.00* (0.00)	−0.00 (0.00)	−0.01* (0.00)
Member of formal and informal groups/institutions	0.16 (0.11)	0.60*** (0.18)	0.13** (0.05)	0.17** (0.06)	−0.00 (0.05)	0.25** (0.13)
Adoption of improved cowpea varieties	0.20** (0.10)	0.14 (0.15)	0.09 (0.06)	−0.09 (0.07)	0.21*** (0.06)	0.17 (0.13)
Wealth index	0.12*** (0.03)	0.17*** (0.05)	0.03** (0.02)	0.07*** (0.02)	0.06*** (0.02)	0.04 (0.03)
Distance from residence to field	0.00 (0.00)	0.00 (0.00)	0.00** (0.00)	0.00 (0.00)	0.00* (0.00)	0.01** (0.00)
Distance to the local (village) market from residence	0.00 (0.00)	0.00 (0.00)	0.00 (0.00)	−0.00 (0.00)	0.00** (0.00)	0.00 (0.00)
Northwest dummy	0.06 (0.11)	−0.28* (0.17)	0.07 (0.05)	−0.15** (0.07)	0.09 (0.06)	0.03 (0.11)
Constant	7.52*** (0.39)	8.71*** (1.08)	11.09*** (0.25)	11.48*** (0.50)	11.18*** (0.21)	12.57*** (0.84)
Observations	1,288	229	1,288	229	1,288	229
Wald test for endogeneity of treatment variable	*χ* ^2^ (2) = 12.10; *p* > *χ* ^2^ = .00		*χ* ^2^ (2) = 13.01; *p* > *χ* ^2^ = .00		*χ* ^2^ (2) = 5.31; *p* > *χ* ^2^ = .07	

Note

Robust standard errors in parentheses.

* *p* < .10.

** *p* < .05.

*** *p* < .001.
